# Autophagic stagnation: a key mechanism in kidney disease progression linked to aging and obesity

**DOI:** 10.1007/s10157-025-02653-4

**Published:** 2025-03-25

**Authors:** Takeshi Yamamoto

**Affiliations:** Department of Nephrology, Osaka University Graduate School of Medicine, 2-2 Yamada-oka, Box D11, Suita, Osaka 565-0871 Japan

**Keywords:** Kidney aging, Lysosomal dysfunction, Obesity-related proximal tubulopathy, Lipid overload, TFEB

## Abstract

Autophagy, a critical intracellular degradation and recycling pathway mediated by lysosomes, is essential for maintaining cellular homeostasis through the quality control of proteins and organelles. Our research focused on the role of proximal tubular autophagy in the pathophysiology of aging, obesity, and diabetes. Using a novel method to monitor autophagic flux in kidney tissue, we revealed that age-associated high basal autophagy supports mitochondrial quality control and delays kidney aging. However, an impaired ability to upregulate autophagy under additional stress accelerates kidney aging. In obesity induced by a high-fat diet, lysosomal dysfunction disrupts autophagy, leading to renal lipotoxicity. Although autophagy is initially activated to repair organelle membranes and maintain proximal tubular cell integrity, this demand overwhelms lysosomes, resulting in “autophagic stagnation” characterized by phospholipid accumulation. Similar lysosomal phospholipid accumulation was observed in renal biopsies from elderly and obese patients. We identified TFEB-mediated lysosomal exocytosis as a mechanism to alleviate lipotoxicity by expelling accumulated phospholipids. Therapeutically, interventions such as the SGLT2 inhibitor empagliflozin and eicosapentaenoic acid restore lysosomal function and autophagic activity. Based on these findings, we propose a novel disease concept, “Obesity-Related Proximal Tubulopathy.” This study underscores autophagic stagnation as a key driver of kidney disease progression in aging and obesity, offering insights into the pathophysiology of kidney diseases and providing a foundation for targeted therapeutic strategies.

## Introduction

Chronic kidney disease (CKD) has reached epidemic proportions globally, affecting nearly one in five individuals in Japan and posing a significant public health challenge. The rising prevalence of CKD is closely linked to the aging population and increasing rates of obesity, both of which are major contributors to CKD development and progression.

Macroautophagy/autophagy, first observed by pathologists approximately 70 years ago, remained poorly understood for decades regarding its physiological relevance and molecular mechanisms. A breakthrough came in the 1990s with the discovery of autophagy-deficient yeast mutants, which spurred rapid advancements in elucidating its molecular basis. Autophagy is a highly conserved catabolic pathway in eukaryotic cells that facilitates the degradation and recycling of damaged or aged cellular components, thereby maintaining intracellular quality control and homeostasis.

Studies using autophagy-deficient mice have highlighted the crucial role of autophagy in maintaining proximal tubular homeostasis, particularly in protecting against various cellular and systemic stressors. More recently, evidence has emerged that dysregulated tubular autophagy under clinically relevant conditions—such as kidney aging and obesity—contributes significantly to disease pathophysiology.

In this review, we present our findings and perspectives on the role of autophagy in proximal tubule epithelial cells (PTECs), with a focus on its implications for the pathophysiology of kidney diseases.

## What is autophagy?

**Fig. 1:**
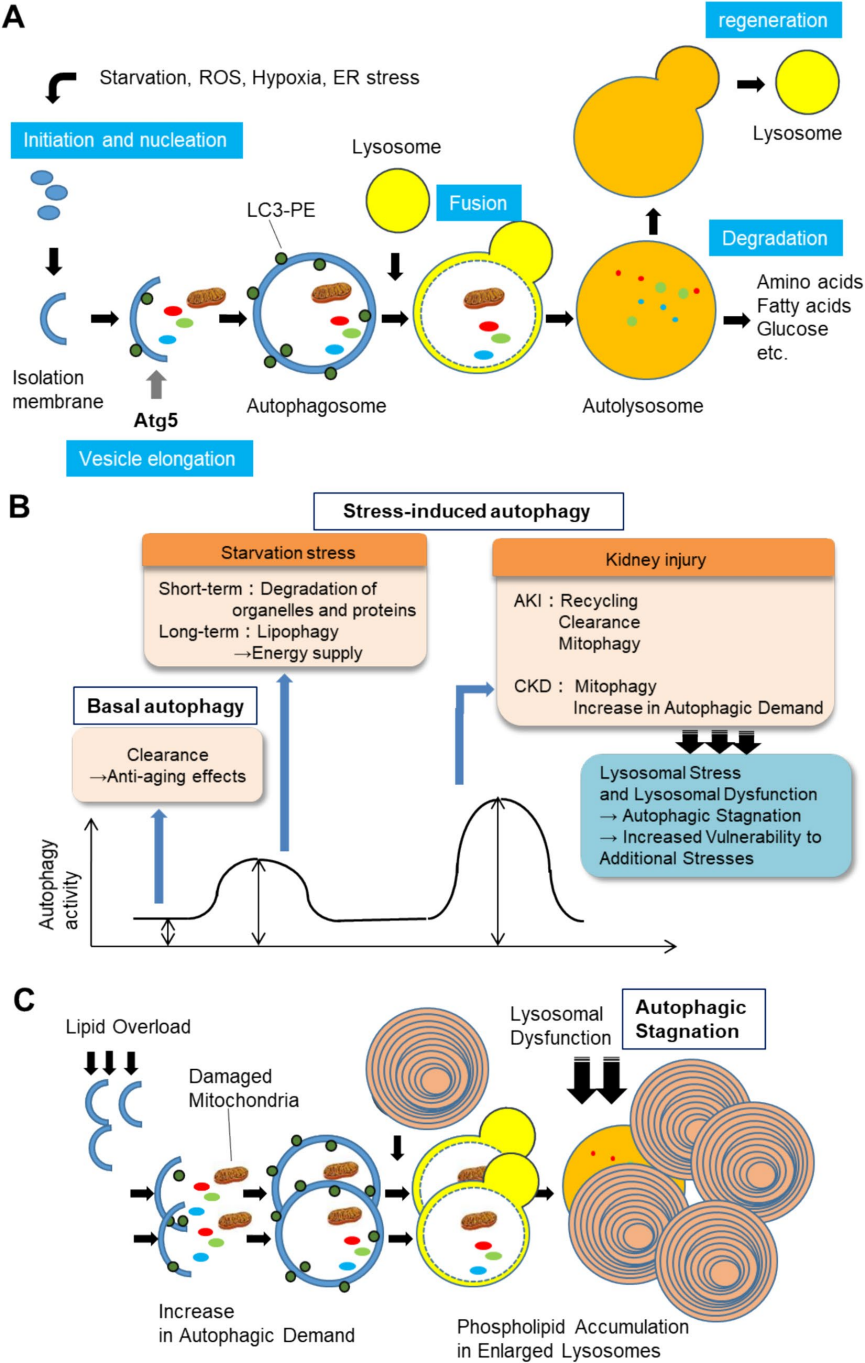
The core machinery of autophagy, regulation, and autophagic stagnation. **A** Upon induction of autophagy, cytoplasmic components are engulfed by a double-membrane organelle called the autophagosome, which subsequently fuses with a lysosome to degrade its contents into recyclable materials. **B** The roles of basal and inducible autophagy under various physiological and pathological conditions (including proposed mechanisms). Basal autophagy serves a clearance function, maintaining cellular homeostasis and exerting anti-aging effects. Inducible autophagy, triggered by short-term starvation stress, facilitates the degradation of organelles and proteins, while long-term starvation activates lipophagy to ensure energy supply through lipid metabolism. During renal injury stress, such as in AKI, autophagy supports recycling, clearance, and mitophagy to mitigate damage. **C** In contrast, during CKD, a significant increase in autophagy demand leads to lysosomal stress and dysfunction, resulting in impaired autophagic flux. These figures were created by the authors, Takeshi Yamamoto, Dr. Yoshitsugu Takabatake, and Prof. Yoshitaka Isaka

The term “autophagy” is derived from the Greek words *auto* (self) and *phagos* (to eat) and refers to a cellular system for degrading cytoplasmic components within the acidic environment of lysosomes [1, 2]. The autophagy process unfolds as follows (Fig. 1a): upon starvation or organelle damage, a flat membrane structure known as the isolation membrane forms in the cytoplasm. This membrane elongates and engulfs damaged organelles and proteins targeted for degradation, creating a double-membrane vesicle called an autophagosome. The autophagosome then fuses with lysosomes to form an autolysosome, where the enclosed contents are degraded by lysosomal hydrolases. The resulting degradation products are recycled for protein synthesis and energy production, while the lysosome is regenerated from the autolysosome.

Structures resembling what we now recognize as autophagosomes and autolysosomes were first observed in 1957 [3]. However, autophagy research remained primarily descriptive and morphological until 1993, when Prof. Yoshinori Ohsumi and colleagues identified autophagy in yeast and generated autophagy-deficient mutants [4]. This breakthrough enabled the discovery of essential autophagy-related (Atg) proteins, many of which are involved in ubiquitin-like conjugation systems essential for autophagy progression. Among these, *Atg5* and *Atg7* knockout or knockdown models are commonly used to study autophagy deficiency (Fig. 1a).

## Methods for observing and evaluating autophagic activity

The fundamental method for detecting autophagosomes morphologically is electron microscopy, which identifies double-membrane structures. The lipidation of LC3 (a homolog of Atg8) to form LC3-II, which localizes to autophagosome membranes, is widely used to visualize autophagosomes with fluorescently tagged LC3 or to assess LC3-II levels via SDS-PAGE (Fig. 1). In addition, GFP-LC3 transgenic mice have been developed to allow in vivo visualization of autophagosomes and are extensively utilized worldwide [5].

Since autophagy is a dynamic process in which autophagosomes are continually formed and degraded, measuring autophagic flux—the rate of this cycle—is crucial. For in vitro experiments, autophagic flux is often estimated by treating cells with autophagy/lysosome inhibitors and evaluating LC3-II accumulation. In 2016, we described a method to estimate autophagic flux in vivo by comparing the number of GFP-positive puncta in GFP-LC3 transgenic mice treated with chloroquine 6 h before euthanasia to untreated controls [6]. The accumulation of SQSTM1/p62, a substrate that interacts with LC3 and is efficiently incorporated into autophagosomes, is another established method for assessing autophagic activity. In autophagy-deficient cells, SQSTM1/p62 accumulates due to impaired degradation, forming aggregates that reflect defective autophagy. Comprehensive guidelines for autophagy assays have been periodically updated under the leadership of Prof. Daniel Klionsky and provide valuable resources for researchers in the field [7].

## Autophagy in the kidney

Baseline autophagic activity varies across organs, with the liver demonstrating high activity, degrading approximately 2% of total protein per hour under starvation conditions [8]. The kidney, particularly the proximal tubule, also exhibits significant autophagosome formation under both basal and stress conditions [9–13]. As early as 1957, inclusion bodies containing small amounts of mitochondria were observed within lysosomes during the differentiation of renal tubular cells in neonatal mice [3]. However, since autophagy regained prominence in the 2000s, numerous studies have reported “increased” autophagy in various tubular injury models. Unfortunately, the physiological significance of these observations remained unclear due to the reliance on nonspecific autophagy inhibitors.

In 2011, our research group generated mice with proximal tubule-specific autophagy deficiency by crossing KAP-Cre mice (expressing Cre recombinase mainly in the S2-3 segments of the proximal tubule) with *Atg5 flox* mice. These knockout (KO) mice developed ubiquitin-positive aggregates and abnormal mitochondria in proximal tubular epithelial cells (PTECs) by 9 months of age [14]. By 2 years, they displayed phenotypes indicative of accelerated kidney aging, including interstitial fibrosis, suggesting that basal autophagy is essential for maintaining proximal tubular homeostasis [6]. Furthermore, using the aforementioned method to monitor autophagy in GFP-LC3 transgenic mice, we demonstrated that age-dependent high basal autophagy mitigates kidney aging, while a diminished capacity to upregulate autophagy under additional stress accelerates the aging process [6].

## Lipophagy: starvation response in proximal tubules

What happens to proximal tubules during starvation, the most fundamental autophagy inducer? In mammals, starvation triggers lipid mobilization in adipose tissue, leading to the release of free fatty acids, which are subsequently stored as lipid droplets (LDs) in peripheral tissues and metabolized via β-oxidation in mitochondria for energy production. Lipids are a critical energy source for PTECs, which have a high energy demand. To investigate the relationship between autophagy and lipid metabolism, we examined the roles of LDs and autophagy in starvation-induced energy metabolism in proximal tubules [15].

Starvation in wild-type mice for up to 48 h resulted in the accumulation of LDs on the basolateral side of PTECs. Some LDs co-localized with the autophagosome marker LC3, and LC3 was abundantly detected in LD fractions. Electron microscopy revealed LDs enclosed by autophagosomes. Pulse-chase assays using fluorescently labeled fatty acids in cultured PTECs confirmed that LDs are transported to lysosomes via autophagosomes for degradation—a process termed lipophagy [16]. Autophagy-deficient cells failed to degrade LDs properly, leading to lipid accumulation, ATP depletion, and increased vulnerability to starvation stress. Similarly, proximal tubule-specific autophagy-deficient mice subjected to 48 h of starvation exhibited significant LD accumulation and reduced β-oxidation compared to wild-type mice. These findings indicate that lipophagy plays a crucial role in maintaining energy homeostasis in PTECs during starvation [15] (Fig. 1b).

## Stagnation of autophagy in proximal tubules during aging and obesity

We sought to investigate whether proximal tubular autophagy is disrupted under stress conditions relevant to clinical settings, rather than in genetically modified mice. Through extensive trial and error, we identified lipid overload as a pertinent stressor [17]. When PTECs were exposed to palmitic acid (PAL), autophagosome formation was initially enhanced. However, over time, the accumulation of SQSTM1/p62-positive protein aggregates was observed, indicating a blockade in the late stages of the autophagic pathway. Electron microscopy revealed abnormal mitochondrial morphology and the accumulation of undigested organelles within lysosomes. Observations using LysoSensor demonstrated impaired lysosomal acidification due to PAL exposure.

In 2016, we generated mice with tamoxifen-inducible, proximal tubule-specific autophagy deficiency by crossing Ndrg1–Cre^ERT2^ mice with *Atg5 flox* mice [6]. Two months of high-fat diet (HFD) loading followed by 3 weeks of tamoxifen-induced autophagy deficiency revealed increased SQSTM1/p62-positive aggregate accumulation in the proximal tubules of the HFD group compared to the normal diet (ND) group. This finding suggests that HFD increases the autophagic workload in proximal tubules. Large vacuoles were observed in the proximal tubules of HFD-fed wild-type mice, identified as lysosomes with LAMP1-positive membranes and intraluminal phospholipid accumulation, which appeared as multilamellar bodies (MLBs) under electron microscopy, rather than LDs.

Pulse-chase assays using cultured cells demonstrated that phospholipids, particularly those from mitochondrial and other organelle membranes, redistributed via autophagic clearance of damaged mitochondria—a process termed mitophagy[18]—accumulating in lysosomes under lipid overload conditions. This indicates that lysosomal dysfunction, accompanied by excessive phospholipid accumulation, leads to impaired autophagic flux. Autophagy is involved in the formation of MLBs, as deletion of *Atg5* prevents tubular vacuolation (phospholipid accumulation in enlarged lysosomes) in HFD-induced obese mice. However, autophagy deficiency severely exacerbates HFD-induced mitochondrial dysfunction, inflammation, and fibrosis. Furthermore, we discovered that the kidneys of the obese mice are unable to activate autophagy to counteract further stress, thereby showing increased vulnerability to acute kidney injury (AKI) caused by ischemia–reperfusion (I/R) injury. These findings suggest that while dependence on autophagy increases under HFD conditions, lysosomal dysfunction hampers smooth autophagic progression, resulting in kidney injury (Fig. 1c).

The phenomenon of “a significant increase in autophagic demand, lysosomal stress and dysfunction, and consequent impaired autophagic flux” is a shared characteristic, albeit with differences, observed in conditions such as kidney aging [6, 19], diabetic kidney disease (DKD) [20, 21], and high phosphate diet mediated-CKD progression [22]. We proposed this common mechanistic phenomenon as “stagnation of autophagy” [23] (Fig. 1c).

We further explored compensatory mechanisms that act against autophagic stagnation [19]. This study demonstrated that impaired autophagy in PTECs during aging or obesity induces fibroblast growth factor 21 (FGF21) production, known for its anti-aging and anti-obesity properties [24]. FGF21 alleviates autophagic stagnation and maintains mitochondrial homeostasis, thereby reducing CKD progression [19].

## Obesity-related proximal tubulopathy (ORT)

Obesity-related kidney disease is typically characterized by glomerular hypertrophy and segmental sclerosis, commonly referred to as “obesity-related glomerulopathy (ORG)” [25]. While much attention has been focused on glomerular lesions, recent evidence indicates that lipid overload-induced tubular lesions, such as MLB accumulation, contribute to kidney dysfunction, inflammation, and fibrosis [17, 26–30].

Obesity is associated with adipocyte hypertrophy, leading to the secretion of pro-inflammatory saturated fatty acids [31]. PTECs are particularly vulnerable to lipid overload, as they actively uptake fatty acids from both circulation and glomerular filtrate via receptors such as LRP2 (low-density lipoprotein receptor-related protein/MEGALIN). LRP2-mediated endocytosis of glomerular-filtered albumin-bound fatty acids is involved in MLB formation, as this process is blocked by *lrp2-/-*deletion in HFD-fed obese mice [28, 32].

**Fig. 2:**
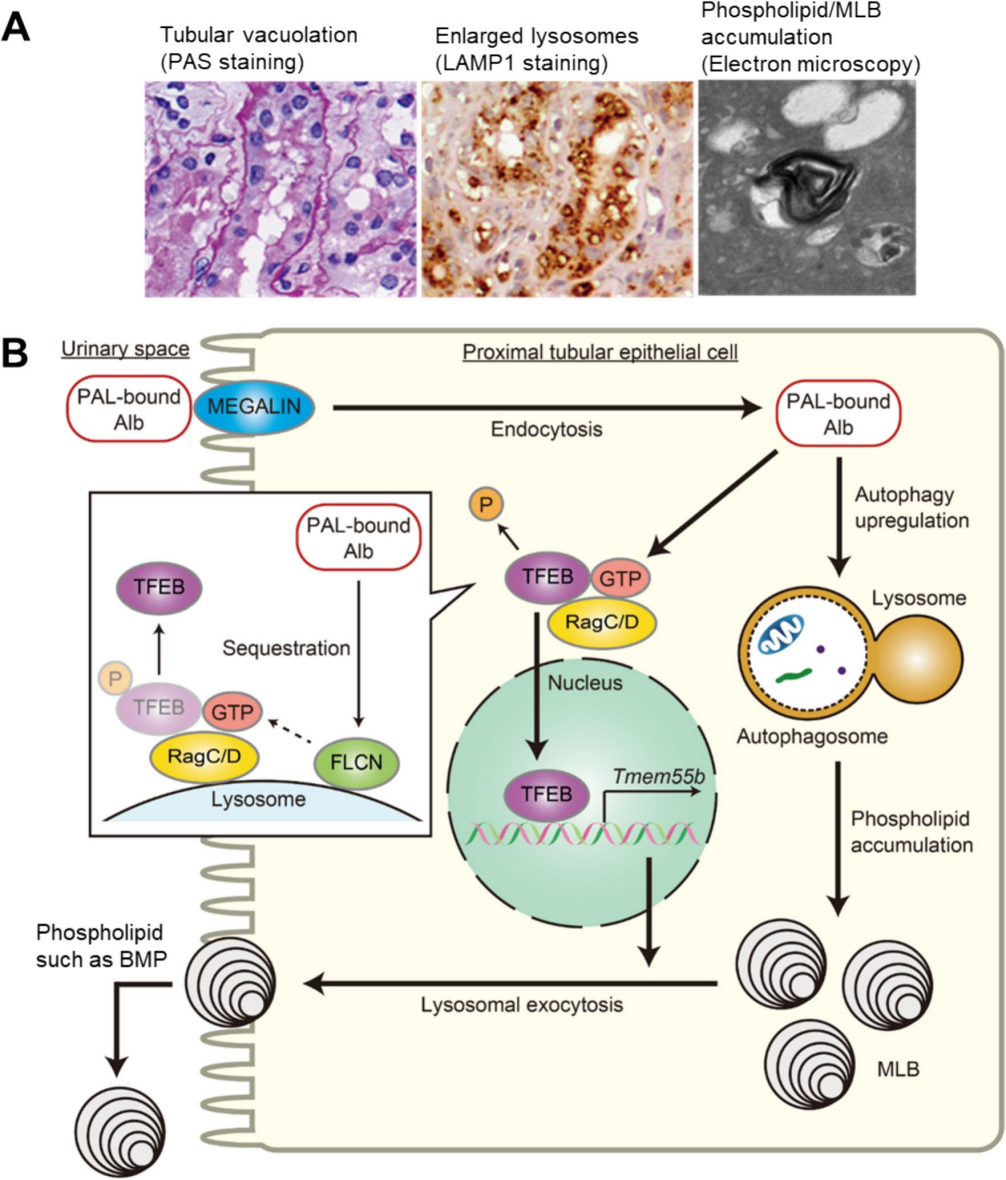
Lysosomal dysfunction and TFEB-mediated counteraction in ORT. **A** Representative images of kidney specimens obtained from obese patients (PAS staining, LAMP1 staining, and electron micrograph). **B** Lysosomal dysfunction leads to autophagic stagnation in obesity-related proximal tubulopathy (ORT), while TFEB-mediated lysosomal exocytosis of phospholipids counteracts ORT. Proximal tubular epithelial cells (PTECs) retrieve albumin-bound palmitic acid (PA) from the glomerular filtrate via LRP2-mediated albumin (Alb) endocytosis. PA induces autophagy, mobilizing phospholipids from cellular membranes into lysosomes, which results in MLBs accumulation. In parallel, PA promotes TFEB nuclear translocation by inactivating RRAG GTPase through the sequestration of folliculin (FLCN) onto the lysosomal membrane. This process mediates lysosomal exocytosis, preventing MLBs accumulation and counteracting lipotoxicity. Reprinted from *Journal of the American Society of Nephrology* (JASN)[17] and *JCI Insight* [33]

In exploring regulatory factors that protect against lipotoxicity, we identified transcription factor EB (TFEB) as a modulator of PTEC lipotoxicity [33]. TFEB regulates the expression of target genes bearing the coordinated lysosomal expression and regulation (CLEAR) motif, thereby regulating lysosomal biogenesis and function [34, 35]. First, lipid overload induces TFEB nuclear translocation via RAG C/D-dependent inhibition of the mTORC1 pathway in PTECs, which prevents phospholipid accumulation in lysosomes by promoting lysosomal exocytosis. Second, in HFD-fed mice, activated TFEB in PTECs counteracted phospholipid accumulation in lysosomes by promoting lysosomal exocytosis of MLB into urine. Conversely, TFEB deficiency resulted in autophagic stagnation and increased vulnerability to I/R injury in obese mice. Third, in CKD patients, higher body mass index correlated with increased tubular vacuolation (phospholipid accumulation in enlarged lysosomes) and reduced TFEB nuclear localization in PTECs. These findings suggest that insufficient TFEB activity, increased tubular vacuolar lesions, and autophagic stagnation are principal determinants of kidney function decline in obese patients (Fig. 2a).

Notably, tubular vacuolar lesions in obesity-related kidney disease, characterized by lysosomal phospholipid/MLB accumulation, can be distinguished from those resulting from LD accumulation [15, 17]. Our observations in mouse kidneys revealed that lysosomal phospholipid accumulation is localized to the apical side of proximal tubules, whereas LD accumulation is found on the basolateral side, adjacent to the mitochondria. Moreover, lysosomal phospholipid accumulation does not stain with Oil-Red O, whereas LDs are readily stained by Oil-Red O. Electron microscopy demonstrates that phospholipid accumulation forms a multilamellar structure, while LDs exhibit a homogeneous structure. Immunohistochemical analysis further differentiates the two: lysosomal phospholipid accumulation is LAMP1-positive, while LDs are positive for adipose differentiation-related protein (ADRP), enabling clear distinction between the two [15, 17]. Collectively, we have proposed a novel disease concept, “Obesity-Related Proximal Tubulopathy (ORT),” as an emerging threat to kidney health (Fig. 2b) [36].

## Therapeutic applications of autophagy

As described above, therapeutic strategies are needed for conditions where autophagic demand increases but the process is impaired. Current efforts aim to harness autophagy for therapeutic purposes, with most approaches focusing on either inhibiting autophagy (e.g., in cancer) or inducing it (e.g., in diseases where autophagy has organ-protective effects). However, in cases of lipid overload, further induction of autophagy may exacerbate the burden on the pathway and worsen the condition [17]. Indeed, pharmacological inducers of autophagy, such as mTOR inhibitors (e.g., rapamycin), have been shown to exacerbate I/R injury in streptozotocin (STZ)-treated type 1 DKD, where autophagy is stagnated due to lysosomal stress [21]. Thus, strategies should focus on preventing autophagic stagnation to address these pathologies.

**Fig. 3:**
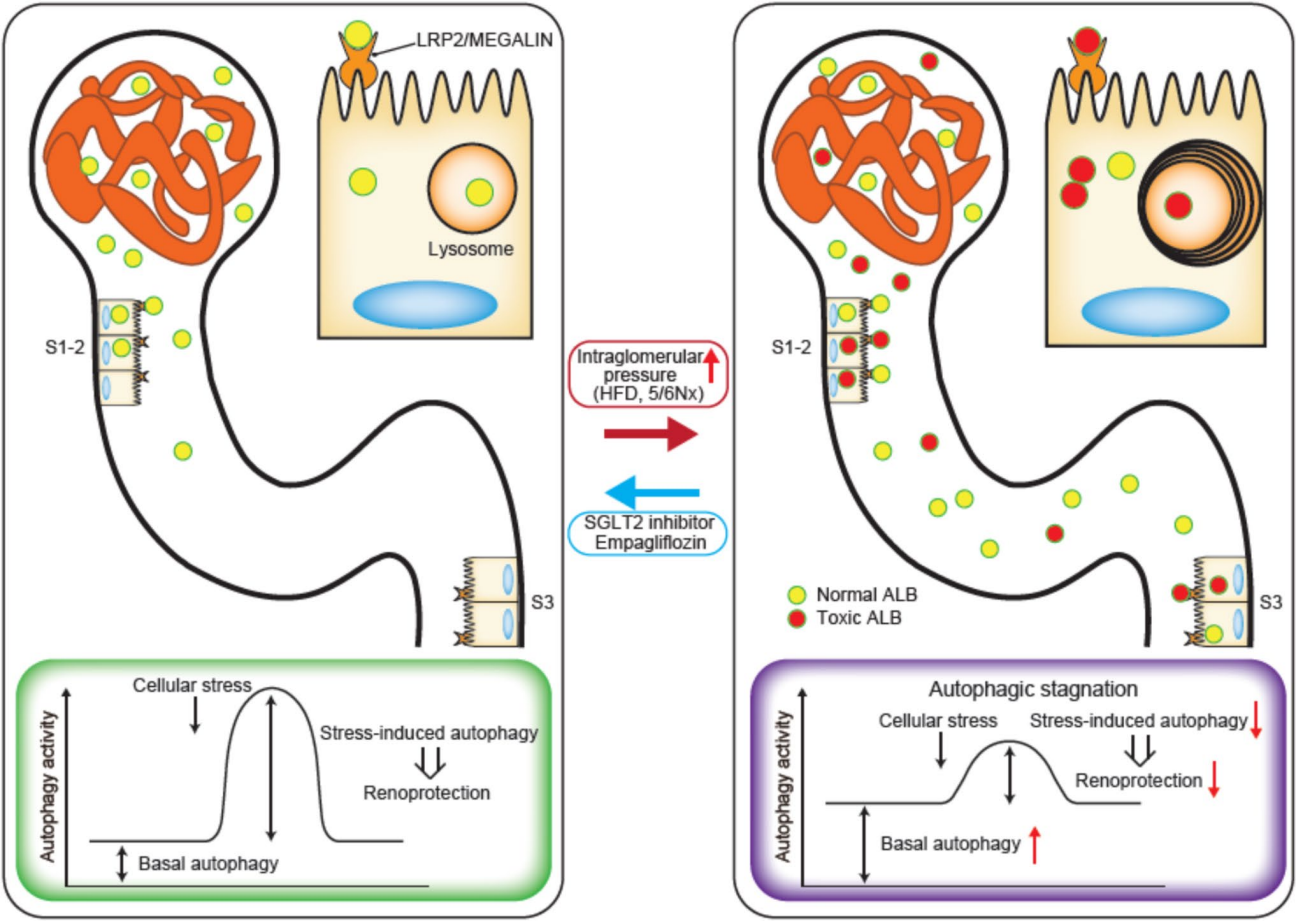
The SGLT2 inhibitor empagliflozin protects the kidney by preventing autophagic stagnation in proximal tubules. Increased glomerular pressure caused by a high-fat diet (HFD) or 5/6 nephrectomy induces tubular reabsorption of toxic albumin via LRP2, leading to increased autophagic demand and stagnation of autophagic flux, which heightens vulnerability to AKI. The SGLT2 inhibitor empagliflozin reduces intraglomerular pressure and ameliorates metabolic dysfunction, thereby improving the quantity and quality of filtered albumin and alleviating autophagic stagnation in PTECs. This prevention of autophagic stagnation ultimately enhances PTEC integrity, resulting in renoprotection. Reprinted from *Autophagy* [32]

Our recent findings indicate that eicosapentaenoic acid (EPA), a widely used clinical agent, mitigates lysosomal dysfunction and restores autophagic flux and cellular function during lipid overload by encapsulating saturated fatty acids within protective LDs [37]. More recently, we investigated the renoprotective mechanisms of the SGLT2 inhibitor empagliflozin with a focus on albumin reabsorption and autophagy in proximal tubules. Empagliflozin, which decreases intraglomerular pressure, not only reduced the HFD-induced increase in albumin reabsorption via LRP2 in the proximal tubules but also ameliorated the HFD-induced imbalance in circulating albumin-bound fatty acids. Consequently, empagliflozin alleviated the HFD-induced increase in autophagic demand and successfully prevented autophagic stagnation in the proximal tubules (Fig. 3) [32].

Furthermore, we discovered that trehalose, which enhances TFEB nuclear translocation, reduces HFD-induced formation of cytosolic vacuoles in PTECs [33]. Thus, activating TFEB to promote lysosomal exocytosis and alleviate autophagic stagnation may represent an attractive treatment strategy for ORT. Given that nuclear TFEB localization in PTECs declines with age in both mice and humans [38, 39], understanding the mechanisms underlying autophagic stagnation and regulating lysosomal biogenesis and function appears to be key for therapeutic approaches for CKD patients [40].

## Perspective and conclusion

The kidney was one of the first organs where autophagy-like structures were identified. Seventy years later, we are finally beginning to understand the pathophysiological significance of autophagy. Although this review did not cover the roles of chaperone-mediated autophagy and microautophagy in the kidney, the regulation of lysosomal quantity and quality at the organismal level, the implications of autophagic stagnation in ferroptosis, cellular senescence, or inflammation in the kidney, or the molecular mechanisms of selective autophagy—particularly mitophagy and lipophagy—these remain challenging questions [41].

To further understand changes in autophagy and leverage this knowledge to treat human diseases, it will be necessary to develop non-invasive methods for monitoring autophagic activity. Urine samples could hold promise for detecting autophagic stagnation in the kidney [12]. For instance, urinary levels of various phospholipids, such as bis(monoacylglycerol)phosphate (BMP)—a lysosomal phospholipid increased in patients with phospholipidosis—are elevated in obese mice (Fig. 2b) [33].

In conclusion, autophagic stagnation constitutes a pivotal driver of kidney disease progression in aging and obesity. Our studies open new avenues for CKD treatment strategies targeting autophagic stagnation. Eight years have passed since Prof. Yoshinori Ohsumi was awarded the Nobel Prize in Physiology or Medicine for his pioneering work on autophagy in 2016. We are hopeful that continued advancements in autophagy research will soon lead to therapeutic applications for kidney diseases, ultimately preventing kidney failure and the need for renal replacement therapy.
